# TASA: Text-Anchored State–Space Alignment for Long-Tailed Image Classification

**DOI:** 10.3390/jimaging11110410

**Published:** 2025-11-13

**Authors:** Long Li, Tinglei Jia, Huaizhi Yue, Huize Cheng, Yongfeng Bu, Zhaoyang Zhang

**Affiliations:** School of Information Engineering, Chang’an University, Xi’an 710064, China

**Keywords:** long-tailed image classification, vision–language models, text prototypes, cross-modal alignment

## Abstract

Long-tailed image classification remains challenging for vision–language models. Head classes dominate training while tail classes are underrepresented and noisy, and short prompts with weak text supervision further amplify head bias. This paper presents TASA, an end-to-end framework that stabilizes textual supervision and enhances cross-modal fusion. A Semantic Distribution Modulation (SDM) module constructs class-specific text prototypes by cosine-weighted fusion of multiple LLM-generated descriptions with a canonical template, providing stable and diverse semantic anchors without training text parameters. Dual-Space Cross-Modal Fusion (DCF) module incorporates selective-scan state–space blocks into both image and text branches, enabling bidirectional conditioning and efficient feature fusion through a lightweight multilayer perceptron. Together with a margin-aware alignment loss, TASA aligns images with class prototypes for classification without requiring paired image–text data or per-class prompt tuning. Experiments on CIFAR-10/100-LT, ImageNet-LT, and Places-LT demonstrate consistent improvements across many-, medium-, and few-shot groups. Ablation studies confirm that DCF yields the largest single-module gain, while SDM and DCF combined provide the most robust and balanced performance. These results highlight the effectiveness of integrating text-driven prototypes with state–space fusion for long-tailed classification.

## 1. Introduction

Real-world image classification often shows a long-tailed class distribution in which a few head classes have many samples while many tail classes have few [1,2,3,4,5,6,7,8]. In such settings, head classes tend to form tighter clusters in feature space whereas tail classes are sparse and noisy. This imbalance leads to poor generalization in rare categories and reduces model robustness in real-world applications [9].

Previous work has mainly focused on the visual side, using methods such as re-sampling, re-weighting [10,11], contrastive learning [12,13], or feature enhancement [14,15]. Yet, when tail samples are very limited, visual improvements alone are not enough to close the semantic gap. Vision–language models such as CLIP [16], BLIP [17], and SigLIP [18] introduce textual supervision, yet simple prompts often provide weak guidance. This weak supervision amplifies head-class dominance and further reduces the separability of tail classes. Recent prompt-tuning research has investigated strategies to adapt vision–language models to scenarios with limited training data. LPT [11] addresses long-tailed recognition by introducing class-specific textual prompts to alleviate head-class bias. In contrast, LoCoOp [19] and GALLOP [20] are primarily designed for few-shot or zero-shot classification, where the prompt parameters are optimized under limited supervision. Although these approaches demonstrate the adaptability of prompt learning, they still depend on trainable prompt embeddings, which tend to overfit scarce samples and require additional optimization for each dataset.

To address this issue, we leverage diverse class-level textual descriptions to construct class-aware anchors while avoiding task-specific parameters. Simple class names or a single template such as “a photo of a [CLASS]” provide only limited supervision, especially for tail categories. Large language models (LLMs) can generate richer descriptions, but their outputs are noisy and inconsistent. To mitigate this variance and stabilize textual supervision, we propose SDM. Given several LLM-generated descriptions and a canonical template, SDM forms a class text prototype by cosine-weighted averaging around the template anchor, as detailed in Section 3.1. SDM is training-free on the text side and uses only a few inference hyperparameters.

**Figure 1:**
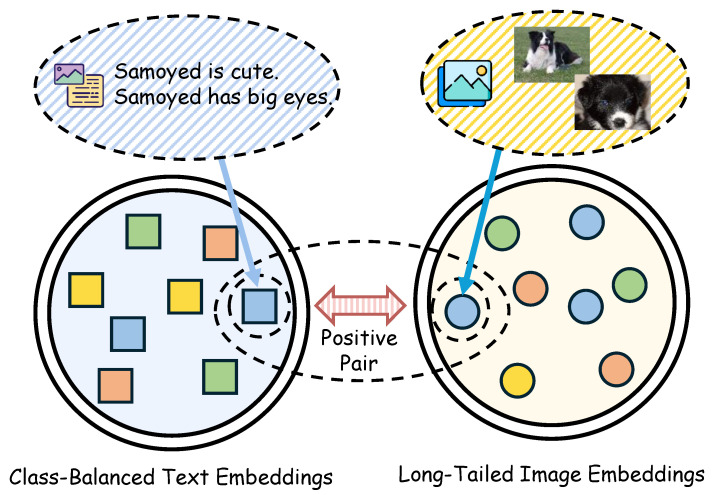
Overview of SDM and DCF with class-aware text prototypes and cross-modal alignment.

To make these anchors effective inside the model, we introduce DCF in Section 3.2. DCF has two branches, one for images and one for text, each with normalization, a linear layer, GELU, and a state–space module implemented with Selective Scan. Cross-modal exchange is realized by conditioning each state–space module on the other branch through learned projections of the other branch’s intermediate features, as shown in Figure 1. After the state–space update, each branch applies element-wise modulation with its residual features. The two outputs are then concatenated and passed through a small MLP to obtain the fused representation. In this way, SDM anchors inform both streams while keeping their own dynamics before alignment.

Figure 1 gives an overview. The blue part on the left denotes the text embedding space and the yellow part on the right denotes the image embedding space. Class-aware text prototypes serve as semantic anchors for long-tailed image features. Aligning each image with its class prototype pulls tail examples toward denser regions and improves cross-modal alignment.

We adopt an end-to-end architecture that integrates SDM, DCF, and alignment in a single forward–backward pass. SDM builds class text anchors on the fly. DCF performs cross-modal fusion. The fused features are mapped to a shared embedding space for alignment and classification. All parts are optimized jointly under one objective and there is no stage-wise training. On CIFAR-10/100-LT [21], ImageNet-LT [3], and Places-LT [22], we observe consistent gains in many-shot, medium-shot, and few-shot groups without paired image–text data or per-class prompt tuning.

In summary, the contributions of this paper are threefold:

- We introduce SDM, which constructs stable class-aware text prototypes from multiple LLM descriptions and a canonical template, providing stronger tail supervision without training text parameters.
- We design DCF, which enables efficient bidirectional interaction between image and text features through state–space modeling and lightweight fusion.
- We evaluate TASA on CIFAR-10/100-LT, ImageNet-LT, and Places-LT, demonstrating consistent improvements across many-shot, medium-shot, and few-shot groups under a unified end-to-end setup.

## 2. Related Work

### 2.1. Long-Tailed Class Learning

Real-world datasets often show a severe class imbalance, known as long-tailed distributions. In these datasets, head classes are easy to learn, while tail classes are rare and difficult to model [1]. To address this, several strategies have been used. These include resampling [10,11] methods that balance the distribution by oversampling the underrepresented classes or undersampling the overrepresented ones. Another approach is reweighting [13,23], where different classes are assigned different importance during training. Data augmentation [24,25] also plays a role by creating new samples for tail classes using geometric transformations, color changes, or added noise [26]. However, a key challenge in data augmentation is not just generating new images, but also making sure that the labels stay semantically correct, which is crucial for improving model performance on tail classes.

### 2.2. Vision–Language and Textual Prototypes

Recent research underscores the substantial influence of prompts on the efficacy of vision–language models. Studies by [20,27] demonstrate that even minor modifications in text, for instance, altering “a photo of [CLASS]” to “a photo of a [CLASS],” can yield notable enhancements in model capabilities [28]. This underscores the critical need for meticulous selection or crafting of effective textual descriptions specific to each task. Nevertheless, the manual identification or creation of ideal prompts is an arduous and protracted endeavor. To address this challenge, researchers have explored various automated or semi-automated approaches for prompt acquisition. A different strategy involves training models to derive prompt representations implicitly from raw data. This can be achieved, for example, through the inclusion of adapter modules or the application of techniques like Prefix or Prompt Tuning [19]. A second significant approach entails employing external tools or models to create effective textual prompts, especially by harnessing the power of large language models (LLMs). Studies like [29] highlight the potential of utilizing LLMs to automate the generation of diverse and potentially more effective VLM prompts. This LLM-based prompt generation approach provides VLMs with a richer and more semantic input view [30], thanks to the conceptual understanding and strong textual organization capabilities of LLMs. CuPL [31] generates class-conditioned textual descriptions with an LLM and uses them as prompts for CLIP, improving zero-shot recognition without retraining. Meta-Prompting [32] automatically generates and selects class-specific prompts via a meta-prompt for stronger zero-shot CLIP classification. Together, these works illustrate LLM-assisted, class-specific prompt generation as a practical way to enhance CLIP-style recognition under limited supervision.

### 2.3. Cross-Modal Alignment and Fusion

Multimodal fusion and cross-modal [33] alignment are closely linked and often used together to improve the performance of cross-modal tasks. Refs.  [16,18,34] used contrastive learning to map images and texts into a shared space. This allows the model to capture relationships between the modalities, improving tasks like image-text retrieval by increasing their correlation and retrieval accuracy. Refs. [17,35] applied a cross-modal attention mechanism to refine feature alignment, strengthening the interaction between image and text features. This approach enhances the model’s ability to perform multimodal reasoning. Recently, refs. [36,37,38] proposed a cross-modal alignment method based on shared embedding spaces. This ensures better alignment of features from different modalities and improves cross-modal reasoning and understanding.

## 3. Method

### 3.1. Semantic Distribution Modulation

**Figure 2:**
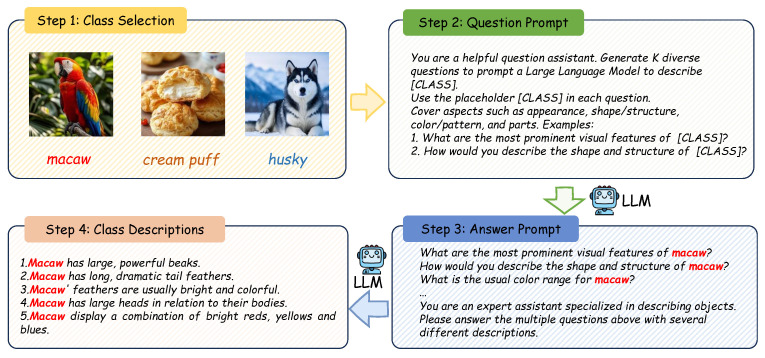
Class-level description generation via LLM Q/A. Step 1: select classes; Step 2: an LLM composes placeholder-based questions with [CLASS]; Step 3: for each class, the LLM answers under an expert-style prompt; Step 4: aggregate answers into multi-attribute descriptions (appearance, shape, color, etc.)

To address the limitations of fixed text templates and the sparsity of tail-class features, we propose SDM. The core idea is to enrich class-level semantics with LLM-generated descriptions, as shown in Figure 2, and integrate them into prototypes via similarity-weighted fusion. These prototypes compensate for missing semantics in tail categories and serve as robust anchors for cross-modal alignment.

#### 3.1.1. Text Data Generation

**Figure 3:**
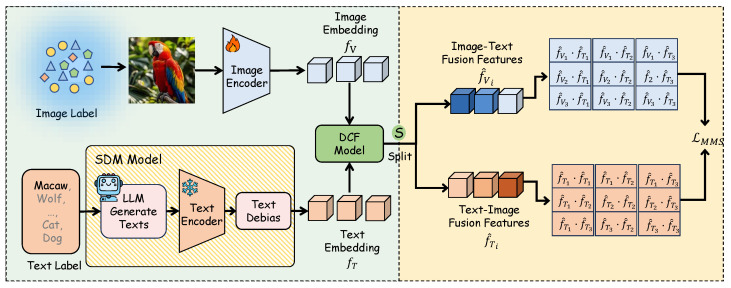
Pipeline of TASA. SDM builds class-balanced text prototypes from LLM-generated descriptions. The image encoder extracts visual features. DCF fuses both modalities to produce ${\hat{f}}_{V}$ and ${\hat{f}}_{T}$, and MMS aligns the fused features in a shared space.

In CLIP-style frameworks, text prompts are typically restricted to a canonical template such as “a photo of a [CLASS]”. Such rigid templates fail to capture diverse real-world attributes, particularly in long-tailed distributions where visual evidence is sparse. To overcome this limitation, SDM adopts a weak pairing strategy, as shown in Figure 3, which leverages LLMs to generate auxiliary class-level descriptions.

For each class *c*, we use a two-step generation process. First, the LLM is prompted to formulate attribute related questions (e.g., color, material, or context) that characterize the key semantics of *c*. Second, it produces descriptive sentences by answering these questions, yielding textual variants that cover both core traits and complementary details. This procedure produces a diverse set of textual representations $\left\{T_{1},T_{2},\ldots ,T_{N}\right\}$, which extend beyond the canonical template and enrich the semantic space with discriminative priors.

#### 3.1.2. Prototype Construction

While LLM-generated descriptions increase diversity, their quality and consistency are not guaranteed. Directly using them may introduce semantic drift. To address this issue, SDM fuses the generated variants with the canonical template through similarity-weighted aggregation, thereby stabilizing the textual space and suppressing noise.

For each class *c*, the canonical template is encoded by the CLIP text encoder to obtain the anchor representation $f_{A}$. The generated descriptions are encoded as $\left\{f_{T_{1}},f_{T_{2}},\ldots ,f_{T_{N}}\right\}$. Their weights are determined by cosine similarity to the anchor:

(1) $$\begin{array}{c} w_{i}=\frac{\exp(\cos\left(f_{A},f_{T_{i}}\right))}{\sum_{j=1}^{N}\exp\left(\cos\left(f_{A},f_{T_{j}}\right)\right)}. \end{array}$$

Here, $w_{i}$ denotes the normalized importance of the *i*-th textual description, reflecting its semantic similarity to the anchor template. Consequently, $w_{i}\in \left[0,1\right]$ and $\sum_{i=1}^{N}w_{i}=1$, where *N* is the number of candidate descriptions for the class and the index *i* enumerates descriptions within the same class. The final prototype is obtained via weighted fusion:

(2) $$\begin{array}{c} f_{T}=\sum_{i=1}^{N}w_{i}\cdot f_{T_{i}}. \end{array}$$

Here, the canonical template serves as a semantic reference to suppress noise, while the diverse variants inject richer contextual cues. The resulting prototype $f_{T}$ is the output of SDM. It provides a balanced, semantically stable text embedding that compensates for visual long-tail bias and serves as input to the DCF module.

### 3.2. Dual-Space Cross-Modal Fusion

**Figure 4:**
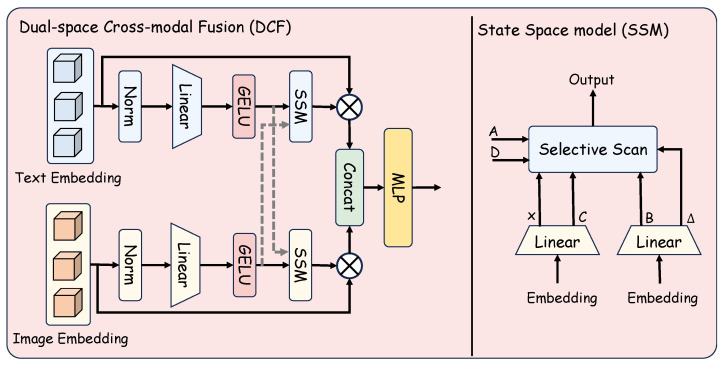
Dual-space fusion: each modality is projected into two spaces, processed by SSMs, then fused via element-wise gating and concatenation, followed by an MLP.

While SDM provides balanced textual prototypes, the visual stream under long-tailed distributions remains sparse and biased toward head classes. We therefore introduce DCF, as shown in Figure 4, to enable efficient bidirectional interaction between image and text features. Built upon selective-scan state–space modeling, DCF performs linear-complexity sequence processing and incorporates two key mechanisms: bidirectional conditioning across modalities and dynamic importance reweighting that improves the separability of tail categories.

#### 3.2.1. State–Space Modeling with Selective Scan

The state–space block is parameterized jointly by textual and visual features. Given the SDM-refined text embeddings $f_{T}\in R^{B\times D}$ and visual embeddings $f_{V}\in R^{B\times D}$, the dynamic parameters are computed as

(3) $$\begin{array}{cc} x,B & =\mathrm{Linear}(f_{T}), \end{array}$$

(4) $$\begin{array}{cc} \Delta ,C & =\mathrm{Linear}(f_{V}). \end{array}$$

The Selective Scan then evolves hidden states according to

(5) $$\begin{array}{cc} h^{m} & =\bar{A}\left(\Delta \right)\;h^{m-1}+\bar{B}\left(\Delta \right)\;x, \end{array}$$

(6) $$\begin{array}{cc} y^{m} & =Ch^{m}+Dx, \end{array}$$

where $\bar{A}\left(\Delta \right)$ and $\bar{B}\left(\Delta \right)$ are functions of the step size Δ. Here, *A* and *D* are learnable matrices that remain fixed during inference, while *B* and *C* are generated dynamically from the input features. In this design, the text branch provides the driving input and mapping parameters, whereas the visual branch contributes the step size and complementary mappings. The step size governs the balance between memory retention and state updates, while the mappings control how new information is injected and read out. This complementary parameterization lets each branch evolve under its own dynamics while being modulated by the other modality, yielding symmetric cross-modal interaction with linear computational complexity.

#### 3.2.2. Fused Outputs for Alignment

After the SSM, we apply a simple fusion readout. For each branch, the SSM output is multiplied by its own input embedding via a skip connection to form a self-gated feature. We then concatenate the two features along the feature dimension and feed them to a shared MLP applied per sample. The MLP outputs two cross-conditioned features, ${\hat{f}}_{T}$ and ${\hat{f}}_{V}$. These fused features are passed to the alignment stage (Section 3.3). Unless noted, ${\hat{f}}_{T}$ and ${\hat{f}}_{V}$ are used for alignment.

### 3.3. Cross-Modal Alignment

Standard cross-entropy contrasts each positive uniformly against all negatives, ignoring class relations. In our setting, class semantics are available from text prototypes. To incorporate class-aware relations, we adopt Margin Metric Softmax (MMS), which augments the logits with prototype distances while maintaining the standard softmax form. This loss aligns each sample with its ground-truth prototype while enlarging margins against semantically distant negatives, thus improving tail discrimination without changing the training pipeline.

Let ${\hat{f}}_{V,i}$ denote the DCF-fused visual feature of sample *i*, and let ${\left\{{\hat{f}}_{T,j}\right\}}_{j=1}^{N}$ denote the fused text prototypes for all classes (here, *i* indexes samples and *j* indexes classes). Write ${\hat{f}}_{T,i}$ as a shorthand for the fused text prototype of the ground-truth class of sample *i*. Define cosine similarity and a text-space distance as

(7) $$\begin{array}{c} S\left(a,b\right)=\frac{a^{⊤}b}{∥a∥\;∥b∥},\;D\left(a,b\right)=1-S\left(a,b\right). \end{array}$$

The per-sample MMS loss is

(8) $$\begin{array}{c} L_{\mathrm{MMS}}=-\log\frac{\exp\;\left(S({\hat{f}}_{V,i},\;{\hat{f}}_{T,i})/\tau \right)}{\sum_{j=1}^{N}\exp\;\left(\left[S\left({\hat{f}}_{V,i},\;{\hat{f}}_{T,j}\right)+\lambda \;D\left({\hat{f}}_{T,i},\;{\hat{f}}_{T,j}\right)\right]/\tau \right)}, \end{array}$$

where τ>0 is the temperature and λ>0 balances the margin term. This loss aligns ${\hat{f}}_{V,i}$ with its ground-truth prototype ${\hat{f}}_{T,i}$ and adds a margin to each negative logit proportional to the prototype distance $D({\hat{f}}_{T,i},{\hat{f}}_{T,j})$, thereby injecting class-aware relations without altering the training pipeline.

## 4. Experiment

### 4.1. Dataset and Evaluation Protocol

In this section, we evaluate our approach using several benchmark long-tailed datasets, namely CIFAR-10-LT, CIFAR-100-LT [21], ImageNet-LT [3], and Places-LT [3]. Following [3,39], the training data is categorized into three refined subsets: many-shot (>100 images), medium-shot (20–100 images), and few-shot (<20 images). Top-1 accuracy is evaluated using each dataset’s validation set.

#### 4.1.1. CIFAR-10/100-LT

As widely utilized benchmarks for long-tailed image classification, CIFAR-10-LT and CIFAR-100-LT are variants of the standard CIFAR-10 (10 categories) and CIFAR-100 (100 categories) datasets, respectively. The original CIFAR dataset contains 50,000 training images and 10,000 test images, each with a size of 32 × 32. The long-tailed versions are created by applying imbalanced sampling to the original training set. The imbalance degree is measured using the imbalance ratio γ, which is calculated as $\gamma =\frac{N_{\max}}{N_{\min}}$, where $N_{\max}$ and $N_{\min}$ correspond to the sample quantities of the most and least common classes, respectively.

#### 4.1.2. ImageNet-LT

ImageNet-LT is a benchmark dataset for long-tailed image classification, derived from the standard ImageNet dataset [3]. Its class distribution adheres to a Pareto distribution (power law), generated by sampling from ImageNet with the power law parameter α set to 0.6. ImageNet-LT consists of 1000 classes and a total of 115K images. The dataset exhibits significant class imbalance, with image counts per class ranging from 1280 for the most frequent class down to 5 for the least frequent. Consequently, its imbalance ratio γ (defined as the ratio of samples in the most to least frequent class) is as high as 256.

#### 4.1.3. Places-LT

Places-LT, a benchmark for long-tailed scene classification [3], is built upon the Places-2 dataset. It comprises 365 classes and 62,500 training images, constructed by sampling according to a natural power law distribution. The dataset exhibits significant class imbalance, with image counts ranging from 4980 (most frequent) to 5 (least frequent). This yields a high imbalance ratio γ of 996, defined as the sample ratio of the most to least frequent class. Conversely, its validation and test sets are balanced, with 20 and 100 images per class, respectively.

### 4.2. Implementation Details

#### 4.2.1. CIFAR-10/100-LT

For CIFAR datasets we begin by constructing a bank of class prompts with GPT-4V [40]. The prompts target shape, color, texture and background context. For each class we sample twenty distinct descriptions, which reduces redundancy and supplies SDM with a diverse textual prior.We use ViT-B/32 as the visual encoder for all CIFAR experiments.

**CIFAR-10-LT.** Training is end-to-end and the CLIP text encoder is frozen. We use a single NVIDIA V100 GPU with a batch size of 32. Optimization uses Adam with a weight decay $5\times 10^{-5}$. The learning rate follows cosine decay for 20 epochs with a warm up of 5 epochs. Two parameter groups are used. The vision encoder decays from $2\times 10^{-5}$ to $2\times 10^{-7}$. The DCF module and the alignment head decay from $1\times 10^{-3}$ to $1\times 10^{-5}$. The MMS temperature is τ=0.05 and the margin weight is λ=0.4. Mixed-precision training with gradient clipping (norm 1.0) and instance-balanced sampling is applied.

**CIFAR-100-LT.** We train the full pipeline under MMS with the CLIP text encoder kept fixed. A single NVIDIA V100 is used with a batch size of 32. The optimizer is Adam with weight decay $1\times 10^{-4}$. Cosine annealing runs for 20 epochs with a 5 epoch warm up. The vision encoder uses $3\times 10^{-5}$ to $3\times 10^{-7}$. The DCF module and the alignment head use $5\times 10^{-4}$ to $5\times 10^{-6}$. We set τ=0.05 and λ=0.5.

#### 4.2.2. ImageNet-LT

ImageNet-LT features a complex long-tailed label space and broad semantic variability. We prepare ten seed prompt templates covering shape, color, texture, and context, adapt them per class, and obtain 10 GPT-4V descriptions per class (API temperature 0.8) [40]. These texts form a candidate bank; during training we build class text anchors on-the-fly by temperature-scaled cosine weighting of their CLIP embeddings. We use ViT-B/16 as the visual encoder for ImageNet experiments.

Training uses four NVIDIA V100 GPUs with a batch size of 32 per GPU for an effective batch size of 128. Training is performed in an end-to-end manner with the CLIP text encoder kept frozen. We adopt AdamW with weight decay $1\times 10^{-4}$. The learning rate follows cosine annealing for 40 epochs and includes a warm up of 10 epochs. Two learning rate groups are used. The vision encoder starts at $5\times 10^{-5}$ and anneals to $5\times 10^{-7}$. The DCF module and the alignment head start at $3\times 10^{-4}$ and anneal to $3\times 10^{-6}$. The MMS temperature is τ=0.07 and the margin weight is λ=0.5.

#### 4.2.3. Places-LT

Scene categories in Places-LT exhibit strong intra-class diversity and fine-grained cues. We curate 10 descriptions per class that explicitly cover spatial layout, typical objects, lighting and contextual cues with GPT-4V (API temperature 0.8) [40], which are then used by SDM to compute similarity weights.We use ViT-B/16 as the visual encoder for Places experiments.

Training is performed on four NVIDIA V100 GPUs with a batch size of 32 on each device, giving an effective batch size of 128. The CLIP text encoder is kept frozen and the rest of the model is optimized end-to-end with AdamW. Weight decay is $2\times 10^{-4}$. The learning rate follows cosine annealing for 40 epochs with a warm up of 10 epochs. The vision encoder uses $3\times 10^{-5}$ to $3\times 10^{-7}$. The DCF module and the alignment head use $2\times 10^{-4}$ to $2\times 10^{-6}$. We set τ=0.10 and λ=0.6.

### 4.3. Overall Results

#### 4.3.1. Results on CIFAR-10/100-LT

Under a unified protocol with the same backbone and training setup, Table 1 summarizes top-1 accuracy on CIFAR-10-LT and CIFAR-100-LT across imbalance ratios 100, 50, and 10. TASA consistently outperforms the MMS [41] baseline on both datasets, with the largest gains on CIFAR-100-LT at IF = 100 and smaller but steady improvements as the imbalance eases (IF = 50, 10). On CIFAR-10-LT, the gains are more modest yet remain positive in all settings, which is consistent with the lower task difficulty and reduced tail sparsity. These results indicate that SDM and DCF are most beneficial under severe imbalance, while remaining effective as class balance improves.

#### 4.3.2. Grouped Results on CIFAR-100-LT

Table 2 presents the top-1 accuracy for the many-shot, medium-shot, and few-shot categories, together with the overall average. Compared with the MMS [41] baseline, TASA achieves notable improvements on the few-shot and medium-shot groups, whereas the change on the many-shot group remains marginal. The overall accuracy increases accordingly. This trend aligns with the objective of TASA, as SDM stabilizes class prototypes when textual information is limited, and DCF strengthens text–image feature fusion when visual evidence is scarce, thereby yielding more balanced performance across head and tail classes.

#### 4.3.3. Overall Results on ImageNet-LT and Places-LT

Table 3 shows the results on ImageNet-LT and Places-LT with the same ViT-B/16 backbone under a unified training setting. TASA performs better than the MMS [41] baseline on both datasets. The gains on ImageNet-LT, which is object-focused, and on Places-LT, which is scene-focused, show that the proposed text-based alignment works across different visual domains. The improvements remain steady despite differences in label detail and image diversity, showing that SDM and DCF are robust beyond the CIFAR datasets.

### 4.4. Components Analysis

#### 4.4.1. SDM Description Count Ablation

**Figure 5:**
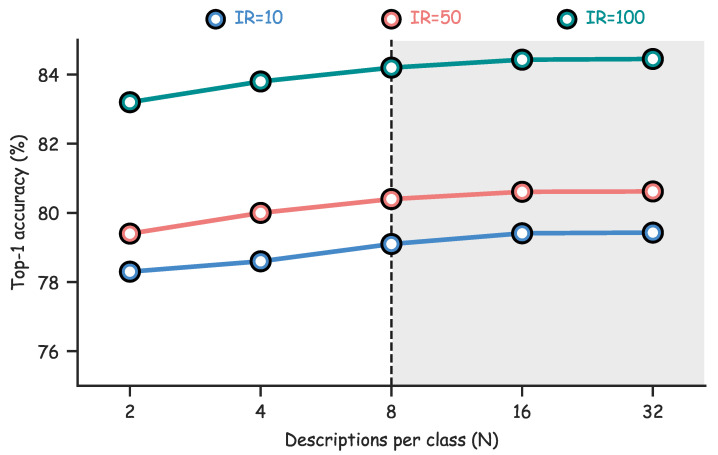
Effect of the number of descriptions per-class on CIFAR-100-LT. Accuracy improves rapidly when N≤8 and saturates for N≥16, indicating that a small yet diverse set of descriptions suffices to stabilize class prototypes.

We evaluate how the number of textual descriptions per class in SDM influences classification on CIFAR-100-LT in Figure 5. The descriptions per class takes the values N∈{2,4,8,16,32} under three imbalance ratios IR=10,50,100. For each setting, SDM forms class text prototypes from the same description pool. The only variable that changes is *N*. The backbone, the training schedule, and all hyperparameters remain fixed across runs.

Top-1 accuracy increases with *N*, with most of the gain achieved when N≤8. Performance improves slightly up to N=16 and then is essentially stable for 16≤N≤32. The pattern is consistent across imbalance levels, and the early gains are more visible under heavier imbalance. These observations indicate that a small and diverse set of descriptions is sufficient to stabilize the text prototypes, and that choosing *N* from 16 to 32 provides a favorable balance between accuracy and efficiency.

The observed trend indicates that the main benefits of SDM arise from the early increase in textual diversity, which broadens the semantic coverage of each class and stabilizes the learned text prototypes. When *N* becomes large, newly added descriptions provide limited new information, and performance gradually drops. The similar behavior under different imbalance ratios suggests that SDM can capture representative semantics for both head and tail categories without requiring an excessive number of descriptions. In practice, choosing N=20 achieves a good balance between accuracy and computational cost.

#### 4.4.2. Module Analysis

As shown in Table 4, on average across the three imbalance levels, DCF improves performance by approximately 2.5 points, SDM by 1.5 points, and their combination by 3.2 points. DCF is the stronger single module, while the pair is best overall. Gains are largest at IR =100 and smaller at IR =50 and IR =10. This pattern shows that both modules mainly help under heavy imbalance.

The joint model stays ahead of the best single module by roughly +0.7 on average. This indicates complementarity rather than duplication. The joint gain remains lower than the sum of the separate gains, which suggests partial overlap. DCF is preferable when computational resources are limited, while combining SDM and DCF yields the most robust results when accuracy is prioritized.

#### 4.4.3. Computational Complexity Analysis

To further analyze the effect of the state–space modeling (SSM) strategy on model complexity and efficiency within the DCF module, we conducted controlled experiments on the base model while keeping all training configurations identical. Two internal variants of the SSM were evaluated, including a linear SSM and a nonlinear SSM. The results are summarized in Table 5, which reports the model parameters, floating-point operations (FLOPs), and average training time per epoch. As shown, the linear SSM introduces a moderate overhead (+10.86 M parameters, +0.67 G FLOPs, 82 s/epoch), while the nonlinear SSM incurs a larger increase (+27.52 M parameters, +1.76 G FLOPs, 109 s/epoch).

From a complexity perspective, the nonlinear SSM introduces several additional sources of computational overhead. First, it replaces the direct linear projection used in the linear SSM with a multi-layer perceptron (MLP) stack that generates the Δ, B, and C matrices in a data-dependent manner. Each of these MLPs contains hidden layers operating on the full feature dimension, thereby increasing both parameter count and intermediate tensor activations. Second, the nonlinear SSM employs gating and nonlinear readout networks (Sigmoid and GELU) to adaptively modulate the output. These operations expand over the hidden dimension and require multiple matrix multiplications, resulting in roughly linear growth in parameters and near-quadratic growth in FLOPs as the hidden width increases. Third, every forward pass involves more kernel calls, memory accesses, and activation computations, which amplify runtime cost beyond the theoretical FLOP ratio. By contrast, the linear SSM uses a single linear mapping with fixed transition matrices, avoiding per-sample conditioning and nonlinear modulation, thus maintaining minimal parameterization and a concise computation graph. Overall, the nonlinear SSM offers greater representational flexibility but at the expense of significantly higher computational complexity, whereas the linear SSM achieves an efficient trade-off and remains the preferred design in our final model.

#### 4.4.4. Text and Image Retrieval with DCF

Flickr30k [57] is a benchmark of natural images with five human captions per image and is commonly used for image–text retrieval. We follow the standard Karpathy split and evaluate zero shot retrieval. Two configurations are run under exactly the same backbone, preprocessing, and evaluation protocol. The visual encoder is ViT-B/16. The first configuration is a frozen CLIP baseline. The second keeps the same backbone and applies our DCF as a training free Top K reranker for both text to image and image to text. The DCF setting shows a consistent improvement, with higher R@1 and other recalls remaining comparable, as shown in Table 6.

On Flickr30k with a frozen backbone and the same setup, text-to-image Recall@1 goes from 58.5 to 58.6, Recall@5 from 83.8 to 84.0, and Recall@10 from 89.1 to 89.4. For image to text, Recall@1 goes from 43.2 to 43.3, Recall@5 stays at 70.4, and Recall@10 goes from 80.4 to 80.5. The gains appear at the top while mid ranks stay steady. This pattern suggests that our head makes the best match stand out without hurting the rest. It first mixes the text and image features, then makes a small normalized tweak to pull the true pair closer. With the backbone frozen and no training, this simple two step design is a good fit and explains the observed lift.

#### 4.4.5. Feature Space Visualization

**Figure 6:**
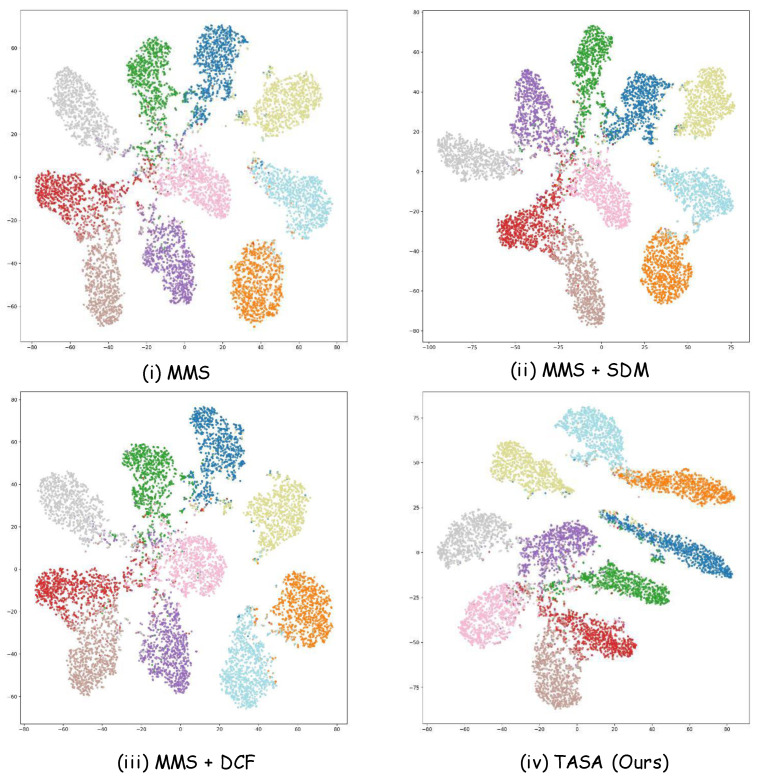
*t*-SNE visualization of feature distributions on CIFAR-10-LT with imbalance ratio 100, illustrating the effect of each module and the final model. Different colors correspond to different classes. MMS serves as the baseline, SDM and DCF progressively improve feature compactness, and the complete TASA model achieves the most distinct and balanced class separation.

We visualize penultimate-layer features using t-SNE [58] on CIFAR-10-LT with an imbalance ratio of 100 to examine the effect of each module and the final model. The comparison includes MMS, MMS+SDM, MMS+DCF, and the complete TASA, all under the same backbone, training schedule, and preprocessing. Features are extracted from the test set and projected into two dimensions using identical t-SNE configurations with a fixed random seed. This visualization reveals how the feature structure evolves as each module is introduced, as shown in Figure 6.

Figure 6 provides a panel-wise comparison on CIFAR-10-LT (IR =100). Panel (i) MMS shows wide, fuzzy clusters with mixing near the center. Adding SDM in panel (ii) opens clearer gaps between several classes, but clusters remain thick and overlaps persist. Replacing SDM with our DCF in panel (iii) contracts the clusters and removes many stray points, with minority classes becoming notably denser. The full TASA model in panel (iv) yields the tightest clusters and the cleanest margins, with little cross-class mixing.

These transitions explain the effect of each component. SDM mainly enlarges between-class distance, hence the wider gaps from (i) to (ii). DCF is a dual-interaction fuse-then-align head; it reduces within-class variance and suppresses outliers, producing the compact islands seen from (ii) to (iii). Combining SDM, DCF, and semantic descriptions stabilizes both head and tail classes, giving the well-separated structure in (iv), which aligns with the accuracy gains reported elsewhere.

## 5. Conclusions

We have presented TASA, an end-to-end framework for long-tailed vision–language classification that mitigates cross-modal imbalance. TASA consists of three main components. SDM aggregates multiple LLM-generated descriptions with a canonical template to form stable class prototypes. DCF employs selective-scan state–space modules to enable bidirectional interaction between image and text branches. MMS performs class-aware alignment by injecting prototype distances into the softmax logits. Under matched settings with a frozen text encoder and a fixed number of descriptions per class, TASA consistently outperforms strong CLIP-based baselines. Ablation studies show that SDM and DCF provide complementary improvements, and further analysis indicates better calibration and more balanced performance across head and tail classes.

TASA also has limitations. In prototype construction, using CLIP text anchors may introduce a mild bias toward CLIP’s embedding space; however, since CLIP is used only as a frozen prior with adaptive weighting, this influence remains limited. Its effectiveness depends on the quality and coverage of the description pool, and DCF introduces additional computation and latency compared with pure contrastive baselines. The current study focuses on classification, while extension to detection, segmentation, and open-vocabulary retrieval is left for future work. Future directions include adaptive description generation and selection, lightweight fusion designs such as attention–state–space hybrids, and theoretical analysis of prototype variance and alignment margins to enhance robustness under extreme long-tailed settings.

## Figures and Tables

**Table 1:** Top-1 accuracy (%) on CIFAR-10-LT and CIFAR-100-LT with imbalance ratio (IR) = 100, 50, 10. Results above the grey line are from prior methods; rows below use the same backbone (CLIP ViT-B/32) for fair comparison. Best results are highlighted in bold and “–” denotes not reported.

Method	CIFAR-100-LT			CIFAR-10-LT		
	**100**	**50**	**10**	**100**	**50**	**10**
Focal Loss [42]	38.4	44.3	55.8	70.4	76.7	86.7
CB-Focal [43]	39.6	45.3	58.1	74.6	79.3	87.1
LDAM-DRW [4]	42.0	46.7	58.6	77.0	81.0	88.1
BBN [44]	42.5	47.0	59.1	79.8	81.2	88.3
MetaSAug [45]	48.0	52.3	61.3	80.6	84.3	89.7
ResLT [46]	48.2	52.7	62.0	82.4	85.1	89.6
BCL [13]	51.9	56.3	64.3	84.3	87.3	90.9
Remix [47]	41.9	49.5	59.4	75.3	–	88.2
logit Adj. [48]	50.5	54.9	64.0	67.2	87.1	90.9
FUR [7]	50.9	54.1	61.3	83.4	86.1	90.7
Proco [5]	52.8	57.1	65.5	85.9	88.2	91.9
CLIP (ViT-B/32)						
CE [43]	67.5	68.1	70.4	91.2	90.0	91.6
BalCE [23]	67.6	68.8	70.8	91.3	91.6	92.4
Focal [42]	66.9	68.6	70.4	89.8	90.0	91.6
LDAM [4]	70.5	72.1	77.2	89.7	91.5	94.4
MMS [41]	75.2	77.5	82.0	93.3	94.5	94.4
TASA (Ours)	**79.4**	**80.6**	**84.4**	**94.1**	**94.9**	**94.6**

**Table 2:** Top-1 accuracy (%) on CIFAR-100-LT, reported for many-shot, medium-shot, few-shot groups and the overall average. Results above the grey line are from prior methods with different settings, while rows below the grey line are our re-implementations under a unified backbone (CLIP ViT-B/32) for fair comparison. Best results are highlighted in bold.

Method	Many	Med	Few	All
RIDE [49]	68.1	49.2	23.9	48.9
DRO-LT [50]	64.7	50.2	23.8	47.3
Logit Adj. [48]	67.2	53.1	32.2	51.9
BCL [13]	67.2	52.9	32.2	51.9
CLIP (ViT-B/32)				
CE [43]	79.3	60.3	61.4	67.5
BalCE [23]	74.6	69.8	57.4	67.6
LDAM [4]	81.6	70.4	58.1	70.5
MMS [41]	**90.3**	75.2	58.1	75.2
TASA (Ours)	87.7	**80.3**	**68.9**	**79.4**

**Table 3:** Top-1 accuracy (%) on ImageNet-LT and Places-LT. The first block lists supervised long-tail baselines, and the second block lists CLIP-based methods with ViT-B/16. TASA uses the same backbone and attains the best accuracy on both datasets. Bold indicates the best; the horizontal grey line is used to separate previous methods from our unified-backbone setting; “–” denotes not reported.

Method	ImageNet-LT	Places-LT
GCL [51]	54.9	–
RIDE [49]	54.9	56.4
logit Adj. [48]	55.1	56.5
BCL [13]	56.0	56.7
Proco [5]	57.3	58.0
CLIP (ViT-B/16)		
TADE [52]	58.8	40.9
MARC [41]	52.3	38.4
GLMC [53]	57.2	–
LiVT [54]	63.8	42.6
SHIKE [55]	59.7	41.9
MMS [56]	70.2	46.3
TASA (Ours)	**72.6**	**47.7**

**Table 4:** Ablation on SDM and DCF with MMS as the baseline. A ✓ indicates the module is used.

SDM	DCF	MMS	IR 100	IR 50	IR 10
		✓	75.2	77.5	82.0
✓		✓	77.3	78.9	83.1
	✓	✓	78.5	79.9	83.9
✓	✓	✓	79.4	80.6	84.4

**Table 5:** Comparison of model complexity, computational cost, and training efficiency across different DCF configurations. The linear and nonlinear SSM variants are built on the same base model (ViT-B/32) and measured under identical training settings for CIFAR-100-LT. “+” denotes that the corresponding component (e.g., DCF or SSM variant) is additionally enabled in that configuration.

Variant	Parameters (M)	FLOPs (G)	Training Time (s/epoch)
BaseModel (w/o DCF)	59.29	188.61	49
+DCF (Linear SSM)	+10.86	+0.67	82
+DCF (Nonlinear SSM)	+27.52	+1.76	109

**Table 6:** Text/image retrieval results. We evaluate performance on the Flickr30k [57] dataset, reporting Recall@1 (%, R@1), Recall@5 (%, R@5), and Recall@10 (%, R@10) for both text and image retrieval tasks. CLIP is a frozen baseline. DCF is a training-free reranker.

Method	Text Retrieval			Image Retrieval		
	**R@1 **	**R@5**	**R@10**	**R@1**	**R@5**	**R@10**
CLIP	58.5	83.8	89.1	43.2	70.4	80.4
CLIP + DCF	58.6	84.0	89.4	43.3	70.4	80.5

## Data Availability

The original data presented in the study are openly available in [CIFAR] at [https://www.cs.toronto.edu/~kriz/cifar.html (accessed on 7 July 2025)]; [ImageNet] at [ http://www.image-net.org/download-images (accessed on 17 July 2025)]; [Places] at [http://places2.csail.mit.edu/ (accessed on 17 July 2025)].
